# Spinal versus general anesthesia in gynecologic laparoscopy: A prospective, randomized study

**DOI:** 10.4274/tjod.galenos.2020.28928

**Published:** 2020-10-02

**Authors:** Berna Kaya Uğur, Lütfiye Pirbudak, Ebru Öztürk, Özcan Balat, Mete Gürol Uğur

**Affiliations:** 1 Gaziantep University Faculty of Medicine, Department of Anesthesiology and Reanimation, Gaziantep, Turkey; 2 Gaziantep University Faculty of Medicine, Department of Obstetrics and Gynecology, Gaziantep, Turkey

**Keywords:** Gynecologic laparoscopy, general anesthesia, spinal anesthesia, oxidative stress, patient satisfaction

## Abstract

**Objective:**

To compare spinal anesthesia (SA) with general anesthesia (GA) in gynecologic laparoscopic surgery regarding anesthetic parameters and patient satisfaction together with an assessment of total oxidant, antioxidant levels, and Oxidative Stress index (OSI).

**Materials and Methods:**

Sixty patients who were planned to undergo gynecologic laparoscopy were randomized into group G (GA) and group S (SA). Demographics, adverse events and anesthetic parameters were recorded before induction, after induction, and at the 5^th^, 10^th^, 15^th^, 30^th^, 60^th^, 90^th^, and 120^th^ minutes. Patients and surgeons completed questionnaires. Total antioxidant capacity (TAC), total oxidant level (TOL), and OSI were measured.

**Results:**

There was no difference between the groups in terms of hemodynamic parameters except heart rate at 30^th^ minute and mean arteral pressure at 10^th^, 15^th^, 30^th^, and 60^th^ minute (p<0.05). The postoperative arterial blood pH value was lower in group S (p=0.021). Intraoperative hypotension was lower in group S (p=0.038). There was more intraoperative hypotension in group S when compared with group G (p=0.038). Postoperative analgesic consumption was higher and onset of postoperative pain was shorter in group G (p=0.001 for both). There was no difference between the groups in terms of patient and surgeon satisfaction. There was no difference in terms of TAC, TOL, and OSI between the groups (p=0.862, p=0.940, and p=0.728, respectively).

**Conclusion:**

SA may become a reliable alternative to GA in gynecologic laparoscopy when hemodynamic and respiratory parameters, patient and surgeon satisfaction, as well as total oxidant, antioxidant levels, and OSI are considered.

**PRECIS:** Spinal anesthesia may be a reliable alternative in gynecologic laparoscopy when anesthetic parameters, patient and surgeon satisfaction as well as total oxidant, antioxidant levels, and oxidative stress index are considered.

## Introduction

Laparoscopic procedures are commonly described as ‘minimally invasive’ and the word minimal is attributed to surgical trauma, pain, hospitalization interval, as well as surgery-induced stress^(1)^. Traditionally, general anesthesia (GA) with controlled ventilation is accepted as the safest technique for laparoscopic procedures, and various myths and dogmas discouraged the use of regional anesthesia, whereas the no anesthetic technique has been proved to be clinically superior to another^(1)^. Possible adverse effects due to pneumoperitoneum or the Trendelenburg position are among the main concerns regarding neuroaxial anesthetic techniques^(2)^.

The stress response is formed due to both anesthetic and surgical interventions via several endocrine and metabolic changes^(3)^. Assessment of oxidative stress is one of the major indicators of the stress response^(4)^. Regional anesthesia is also ‘minimally invasive’ from the anesthetists’ perspective and is currently preferred in many surgical procedures. Many papers have been published regarding the performance of laparoscopic procedures under spinal anesthesia (SA)^(5,6)^.

Theoretically, combining a minimally invasive surgical procedure with a minimally invasive anesthetic technique might appear to lessen oxidative surgical stress that can be measured by oxidative stress markers. We aimed to compare SA with GA in gynecologic laparoscopic surgery regarding safety, patient tolerance, and anesthetic parameters, together with assessment of total oxidant, antioxidant levels and Oxidative Stress index (OSI).

## Materials and Methods

The randomized prospective study was performed at the Department of Anesthesiology and Reanimation of a tertiary health care provider university hospital after approval of the Faculty Ethics Committee (no: 02-2009/46). The study was performed in accordance with the ethical standards described in an appropriate version of the 1975 Declaration of Helsinki, as revised in 2000.

Sixty patients aged between 18 and 45 years who were planned to undergo diagnostic laparoscopy combined with hysteroscopy for unexplained infertility with American Society of Anesthesiologists (ASA) I-II physical status, were enrolled in the study. Randomization was performed with a sealed envelope method and patients were randomized into two groups. None of the patients had premedication. Patients who had more than ASA II physical status, coagulation disturbance, were aged younger than 18 years or older than 45 years, who refused SA, who were cigarette smokers, patients with body mass index (BMI) >30 kg/m^2^, and patients with conversion to laparotomy were excluded from the study. Written consent was obtained from all patients prior to their inclusion in the study.

**Group G: General anesthesia group.** Pre-oxygenation with 100% O_2 _via face mask for 3 minutes, induction with propofol 2 mg/kg, atracurium 0.5 mg/kg, 0.5-1 µg/kg fentanyl and for the maintenance sevoflurane 2-3% and O_2_-air mixture 50%. Additional doses of fentanyl (25 µg) were administered intravenously if patients had tachycardia, sweating, and hypertension due to inadequate surgical analgesia. Sevoflurane (Sevoflurane^®^, AbbVie, United Kingdom) 2-3% + O_2_-air mixture 50% was stopped at last dermal suture. Patients were decurarized with neostigmine 0.06 mg/kg (Neostigmin^®^ Biosel, Istanbul) at the end of the procedure. Patients were extubated when standard extubation criteria were maintained.

**Group S: Spinal anesthesia group. **All patients were informed about the details of the SA procedure - hydroxyethyl starch 6% (5 mL/kg) (Voluven^®^ Fresenius Kabi) for avoiding hypotension due to spinal blockage and 3 L/kg 100% O_2_ with nasal cannula. After stabilization of hemodynamic parameters, patients had SA performed in L_2-3_ with a 25-G Quincke spinal needle (Spinocan^®^ Braun, Germany). Heavy bupivacaine 0.5% 10 mg (2 mL) with fentanyl 25 µg (0.5 mL) was injected to the subarachnoid space. The level of sensorial blockage was tested using a pinprick test. After achieving sensorial blockage at the level of T_4_, patients had 1 mg midazolam intravenously. Saline (5-10 mL/kg/h) was infused during the procedure. Patients who had shoulder pain or surgical pain had 25-75 µg additional fentanyl doses intravenously and sedation was deepened with additional doses of midazolam intravenously. Despite medical treatment, patients who had persistent pain and agitation, conversion to GA as the same way performed in group I and excluded from the study.

Adverse events such as tachycardia, bradycardia, hypotension, hypertension, and conversion to laparotomy were recorded and treated accordingly, if present, in both groups. Also, intraoperative nausea/vomiting (N/V), shoulder pain, agitation, arise of blockage level, and conversion to GA was recorded in group S.

The same anesthetic and surgical teams (the authors of the study) performed spinal or GA, and laparoscopic surgery, respectively.

Patients had standard monitoring, after venous cannulation with 18 G at the dorsum of the hand, including electrocardiogram (5 channel), SpO_2_, non-invasive blood pressure, heart rate, systolic arterial pressure, diastolic arterial pressure, mean arteral pressure, and only for the patients in group S, end-tidal CO_2_ pressure (PETCO_2_) were recorded before induction, after induction, at the incision, at the 5^th^, 10^th^, 15^th^, 30^th^ minutes, and then every 30 minutes. Arterial blood sampling was performed preoperatively from all patients at room air, and also from the patients in group S in room air after the procedure was completed.

All surgeries were scheduled for the early follicular phase of the infertile patients after menses. The procedure was performed in a standard low lithotomy position. Patients were cleaned with 10% povidone-iodine solution and a sterile Foley catheter was inserted after anesthesia. Access into the abdomen was accomplished with closed Veress-needle entry technique after a transumbilical vertical incision and insufflation of carbon dioxide gas up to a pressure of 18 mm Hg was preferred for adequate pneumoperitoneum. We inserted a primary 12-mm trocar at the umbilical incision, as well as other two ipsilateral 5-mm trocars for surgery. A 10-mm 0° laparoscope and operating instruments were inserted through the trocars. The working pneumoperitoneum pressure was lowered to 12 mm Hg after the introduction of all trocars. A Trendelenburg position of no more than 20° was used in both groups. Hysteroscopy was performed in the supine position with 5-mm 30° office hysteroscope using the “no-touch technique” with saline solution as the distention medium.

All patients were connected to a patient-controlled anesthesia (PCA) device (CADD-Legacy^®^ PCA, Smiths Medical MD, Inc. St. Paul, MN, USA) at the end of the procedure. Infusion solution containing 300 mg of tramadol hydrochloride (tramadol HCl) (Contramal^®^ Abdi İbrahim, İstanbul) and 3 mg of metamizole sodium (Novalgin^®^ Aventis, İstanbul) was completed to 100 mL with sterile saline. PCA was set to deliver a bolus of 5 mL with a lockout interval of 15 minutes and 4-hour maximal dose of 20 mL (tramadol HCl 3 mg/mL + 0.03 mg/mL metamizole sodium).

All postoperative adverse events including N/V, sore throat, hoarseness, backache, hypotension, tachycardia, bradycardia, headache, hypertension, itching, itching, desaturation, and transient neurologic symptoms were recorded and treated appropriately.

Time of first mobilization (hour), time of passage of gas or stool (hour), onset time of postoperative pain (min) and postoperative analgesic consumption were recorded. Both the patients (24 hours after procedure) and surgeons (at the end of the procedure) completed simple questionnaires regarding satisfaction including three questions to provide comments about the operation (appendix I and II) adapted from Yuksek et al.^(7)^.

Antecubital venous blood samples for assessment of total oxidant, antioxidant levels and OSI of 6 mL were obtained from all patients in both study groups at the end of the procedure. All blood samples were centrifuged at 1500^-1^ *g* for 10 min and were finally put into Eppendorf tubes within an hour and sera were stored at -80 °C.

A fully automated method developed by Erel^(8)^ was used for the measurement of total oxidant level (TOL) and total antioxidant capacity (TAC)^(9)^. The novel automated method is based on the bleaching of characteristic color of a more stable ABTS [2.2-Azino-bis(3-ethyl-benzothiazoline-6-sulfonic acid)] radical cation by anti-oxidants. The assay has excellent precision values, which are lower than 3%. The results are expressed as mmol Trolox equivalent/L. Oxidants present in the sample oxidized the ferrous ion-o-dianisidine complex to ferric ion. The oxidation reaction was enhanced using glycerol molecules abundantly present in the reaction medium. The ferric ion produced a colored complex with xylenol orange in an acidic medium. The color intensity, which could be measured spectrophotometrically, was related to the total amount of oxidant molecules present in the sample. The assay was calibrated with hydrogen peroxide and the results are expressed in terms of micromolar hydrogen peroxide equivalent per liter (mmol H_2_O_2_ equivalent/L). The ratio of TOL to TAC was accepted as the OSI. For calculation, the resulting unit of TAC was changed to mmol/L, and the OSI value was calculated according to the following formula: OSI (arbitrary unit) =TOL (mmol H_2_O_2_ equivalent/L)/TAC (mmol L Trolox equivalent/L)^(10)^.

### Statistical Analysis

The normality of distribution of continuous variables was tested using the Shapiro-Wilk test. Student’s t-test (for normal data) and the Mann-Whitney U test (for non-normal data) were used for the comparison of two independent groups, and Wilcoxon tests were used to compare numerical variables measured at two different time points. Pearson’s chi-square test was used for testing relationships between categorical variables.

Mean ± standard deviations or median (minimum-maximum) are given as descriptive statistics for numerical variables. This is a pilot study and therefore power analysis was not performed. Statistical analysis was performed using the SPSS for Windows version 22.0 software and a p-value <0.05 was accepted as statistically significant.

## Results

A total of 60 patients were enrolled in the study and there were 30 patients in both groups (G and S). There was no significant difference between the two groups in terms of age, BMI, ASA risk status, indications for laparoscopy, and surgical and anesthesia duration (Table 1).

The hemodynamic parameters of the patients at baseline, 0, 5^th^, 10^th^, 15^th^, 30^th^, 60^th^, 90^th^, and 120^th^ minutes are shown in Table 2.

There was no significant difference between the groups in heart rate except at the 30^th^ minute, at which heart rate was significantly lower in group S (p=0.01). Mean arterial pressure (MAP) at 10^th^, 15^th^, 30^th^, and 60^th^ minutes was significantly lower in group S. There was no significant difference between the two groups in SpO_2_ values.

PETCO_2_ values of patients in group G at baseline, and the 0, 5^th^, 10^th^, 15^th^, 30^th^, 60^th^, 90^th^ and 120^th^ minutes were 32.73±4.58, 32.77±4.55, 33.37±4.00, 33.90±3.64, 34.80±3.68, 35.30±3.51, 35.58±3.69, 33.71±3.35, and 35.25±2.75 mm Hg, respectively.

Arterial blood gas (ABG) analyses of the patients are shown in Table 3.

There was no rise in sympathetic blockage above T_4_, intraoperative surgical pain, and conversion to GA in any patient belonging to group S. There was no conversion to laparotomy and intraoperative desaturation in patients of either group.

Among the other intraoperative adverse events, intraoperative bradycardia was recorded in two patients in group G (6.66%), and three patients in group S (10%) (p=0.640). No patients had tachycardia. Intraoperative hypotension was recorded in two (6.66%) and eight patients (26.66%) in group G and group S, respectively (p=0.038). Hypotension was managed with intravenous saline infusion in all patients except one patient in group S, who also received a single dose of ephedrine intravenous. Intraoperative hypertension was recorded in only one patient (3.33%) in group G and was treated through the deepening of GA.

Among patients of group S, intraoperative N/V was present in two patients (6.66%), agitation in three patients (10%), and shoulder pain in 17 patients (56.6%). An additional dose of fentanyl and deepening of sedation was required in 12 patients who had shoulder pain, but the procedure was completed uneventfully. The remaining five patients (16.66%) reported discomfort and shoulder pain but did not request additional medication.

Postoperative analgesic consumption was significantly higher in group G when compared with group S, 128.00±25.11 mL (512±100 mg tramadol HCl, 3.84±0.75 metamizole sodium) vs. 63.17 ± mL (252.68 ± mg tramadol HCl, 1.89±0.48 metamizole sodium), respectively (p=0.001). Onset of postoperative pain was 8.56±8.13 min in group G and 138.67±41.50 min in group S (p=0.001). There was no statistically significant difference between the group G and S in terms of post-operative passage of gas 10.60±3.59 vs 9.73±2.15 hours (p=0.261) and also postoperative mobilization time 7.87±31.89 vs 8.20±0.66 hours (p=0.261), respectively.

The most common postoperative adverse event was N/V, which was observed significantly more in group G (14 patients, 46.6%) than in group S (four patients, 13.3%) (p=0.005). Thirteen patients (43.3%) had a sore throat and five patients (16.6%) had hoarseness in group G vs none in group S (p=0.001 and 0.02, respectively). Backache was present in four patients (13.3%) in group G and two patients (6.66%) in group S (p=0.389). Postoperative hypotension was detected in two patients (6.66%) in group S vs none in group G (p=0.15). There was only one patient (3.33%) with tachycardia and one patient with headache (3.33%) in group G vs none in group S (p=0.313). There were no postoperative bradycardia, hypertension, itching, desaturation, and transient neurologic symptoms in any patients.

The answers to the first question of the patient questionnaire revealed no significant difference in terms of comfort during surgery between the groups. Six and four (20%/13.33%) of the patients in group G and S, respectively, evaluated their comfort as “very good”, 15 and 17 (50%/56.66%) as “good”, and nine and six (30%/20%) evaluated the comfort of the operation as “moderate” and 0/3 (0/10%) as “poor” (p=0.248). Twenty-eight (93.3%) of patients in group G and 27 (90%) in group S were pleased after the operation (p=0.640). Twenty-nine (96.6%) patients in group G and 27 (90%) in group S would recommend this operation to others (p=0.301).

In the results of the questionnaire conducted for surgeons, abdominal relaxation of patients undergoing SA was evaluated as “good” for 20 (66.66%) patients and “moderate” for 10 (33.33%) patients. The surgeons stated about whether they had any technical problems arising from SA, “a lot” for none, “a little” for 4 (13.33%), and “none” for 26 (86.66%) patients. In the question of regarding whether there was a surgical difference in the operation of patients who underwent GA and those who underwent SA, surgeons stated that there was no difference in 29 (96.66%) patients.

**Figure 1:**
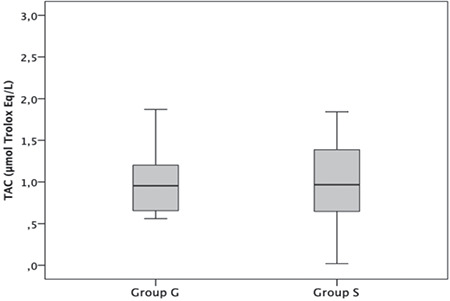
Total antioxidant capacity (TAC) of patients

**Figure 2:**
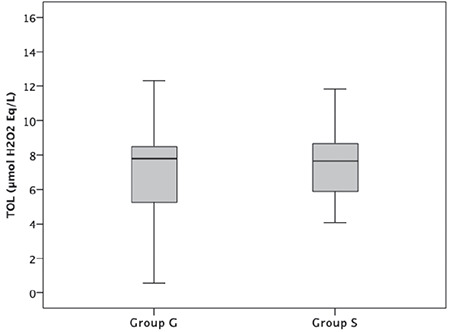
Total oxidant levels (TOL) of the patients

**Figure 3:**
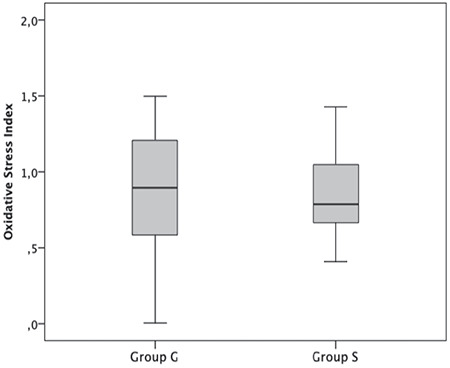
Oxidative Stress index (OSI) of the patients

The results of TAC (Figure 1), TOL (Figure 2), and OSI (Figure 3) are shown in Table 4. There was no significant difference in terms of TAC, TOL, and OSI between the groups (p=0.862, p=0.940, and p=0.728, respectively).

## Discussion

Laparoscopy is a minimally invasive procedure that offers some multiple postoperative benefits including less surgical trauma, pain, pulmonary dysfunction, quicker recovery, and shorter hospital stay^(11)^. There is an increasing trend of preference in favor of laparoscopic procedures compared with laparotomy^(12)^. Traditionally, laparoscopic procedures are performed under GA. Regional anesthesia had not gained popularity in this new era of minimally invasive surgery and not preferred as a first-line choice in gynecologic laparoscopic procedures. According to the literature, regional anesthesia is considered more acceptable as an anesthetic alternative approach in diagnostic laparoscopic procedures of general surgery and laparoscopic cholecystectomies^(1)^. Most studies about SA in laparoscopic surgery involve laparoscopic cholecystectomy, with few cases of appendectomy and hysterectomy^(5,6)^. The main reasons for this withdrawal may be attributed to the fear of adverse effects caused by pneumoperitoneum, which is considered to be not well tolerated by patients who are awake during the procedure, or the Trendelenburg’s position. Data on laparoscopic cholecystectomy do not apply to hysterectomy because the former requires a reverse Trendelenburg position, resulting in more favorable pulmonary dynamics. Conversely, the Trendelenburg position carries concerns regarding pulmonary compliance, making it more challenging to manage the resultant hypercarbia^(13,14,15)^.

An ideal anesthetic method should provide optimal surgical conditions without any physiologic and metabolic harm to the organism, preserve hemodynamic balance, and should also provide prompt and safe recovery in the postoperative period^(16)^. Therefore, a method that provides equable hemodynamic parameters is supposed to be favorable. There was no significant difference between the groups in terms of hemodynamic parameters except heart rate at the 30^th^ minute and MAP at 10^th^, 15^th^, 30^th^, and the 60^th^ minute was significantly lower in group S, which is an expected result due to the sympathetic blockage in SA^(17,18).^

Respiratory parameters regarding SA and GA during laparoscopic surgery are controversial. Spontaneous physiologic respiration during SA has been shown to have a better performance than assisted respiration in GA^(18)^. There were either small or no changes in respiratory function due to mid-thoracic levels of spinal anaesthesia in many studies and alterations in respiratory function that were clinically significant were minimal^(19)^. Also, there were no or little changes in respiratory rate and tidal volume - even with a high level of blockage with SA - and vital capacity decreased slightly^(18)^. Additionally, pulmonary functions return to normal in about 24 hours after laparoscopic procedures performed with GA^(20)^. In our study, respiratory functions were evaluated using ABG analysis. Only the postoperative mean pH value of group S was statistically higher than group G, but still in the physiologic range. This was probably due to hyperventilation of awake patients in group S. Patients in group S could increase the respiratory frequency to lessen the higher pCO_2_ values due to CO_2_ insufflation because they were awake. The mean pCO_2_ values of group S were lower than in group G, which may explain the hyperventilation in this group of patients; however, this was not statistically significant.

A greater increase in PaCO_2_ after CO_2_ pneumoperitoneum when the patient was under GA compared with patients breathing spontaneously was reported, which is similar to our results^(21)^. Sinha et al.^(20)^ reported that there was no significant variation in PaO_2_ or PaCO_2_ during the procedure with SA. Similarly, all ABG parameters were within physiologic limits at the end of the surgery.

Although hypotension in SA seems to be an adverse effect, it is rather a physiologic effect of sympathetic blockage. In addition to SA-related hypotension, the pneumoperitoneum induced rise in intra-abdominal pressure could be another cause for the persistence of hypotension. SA-related hypotension is supposed to be more significant in procedures such as laparoscopic cholecystectomy, which is performed in the Fowler position. Intraoperative hypotension was observed significantly less in patients of group G (6.66%) than in group S (26.6%) (p=0.038), which is also an expected result of SA. In the series of 4.645 patients of Sinha et al.^(20)^, 2.992 underwent laparoscopic cholecystectomy, SA was performed to all patients, and hypotension was observed in 846 (18.21%) patients. This lower frequency of hypotension may be due to lower intraperitoneal insufflation pressure, which was limited to 8-10 mm Hg. In our study, the high-pressure entry technique together with a higher intraperitoneal working pressure during surgery (12 mm Hg) may have contributed to the higher frequency of hypotension in the SA group, but this technique is also safer^(22)^. Intraoperative hypotension frequency is reported between 5.4% and 40%^(22,23,24)^. None of the intraoperative hypotension situations required inotrope support. In our opinion, this may be due to the preoperative colloid administration.

In our study, no adaptation was performed in insufflation or working pressure, which remained constant as 12 mm Hg for the management of intraoperative bradycardia because it was transient without any need for an intervention. Higher intraperitoneal pressures may cause bradycardia due to activation of vagal reflexes and decreasing the insufflation or working pressure in selected cases with persistent bradycardia might improve the situation.

Awake and cooperative patients during laparoscopic procedures might be a preferable situation intraoperatively for communication and quick recovery. Paradoxically, there is a common desire to avoid the performance of SA for laparoscopic procedures. The main reason for this withdrawal is the fear of adverse effects caused by pneumoperitoneum, which is presumably not well tolerated by a patient who is awake during the procedure. Gynecologic laparoscopy in the Trendelenburg position increases infra-diaphragmatic pressure, which may lead to a sensation of pain. We observed neck and shoulder pain in 17 patients (56.66%), of whom 12 (40%) required an additional dose of analgesic. Surprisingly, five (16.6%) of them required no additional dose of analgesia due to the tolerable nature of the pain and all patients completed the procedure uneventfully without conversion to GA. Similarly, in the pilot study of Tzovoras et al.^(25)^, two out of 15 patients had neck or shoulder pain and they were managed with ease. In another randomized controlled study of Tzovoras et al.,^(26)^ pneumoperitoneum pressure was decreased to 10 mm Hg instead of 14 mm Hg and 43% of patients had shoulder pain that received no further treatment or conversion to GA. However, in a study with pneumoperitoneum pressure of 15 mm Hg, 16 patients (55.17%) had shoulder pain^(7)^. Eight patients were managed with intravenous analgesics and three patients needed GA. The remaining five patients had local irrigations of right diaphragmatic crus with local anesthetics^(7)^. In our study, we used 12 mm Hg as the pneumoperitoneum pressure and we did not decrease this pressure in cases of neck and/or shoulder pain without any need for conversion to GA.

In our study, postoperative N/V was troublesome in 14 (46.6%) patients in group G and four (13.33%) patients in group S. This was an expected result due to adverse effects of medications in GA and is consistent with studies reported in the literature^(6,7,20,25)^.

Another major concern that limits the preference of SA in laparoscopic surgery is the comfort and satisfaction of patients. Previous studies based on patient satisfaction reported similar results to ours. Most studies in the literature evaluated only SA and patients were mostly reported as being satisfied^(6,7,20)^.

Questionnaires evaluating patient satisfaction showed that SA was a comfortable alternative anesthetic method. The patients in our study expressed similar comfort during and after the operation with both SA and GA, and they also recommend both anesthetic techniques in a similar fashion. However, the subjective character of this evaluation is a limitation. Theoretically, no patient can compare both anesthetic techniques because no one ever steps in the same river twice.

Inadequate abdominal muscle relaxation is one of the major problems experienced in laparoscopic surgery under SA. This is one of the leading problems reported by surgeons in every abdominal surgical procedure. Surgeons that performed laparoscopic cholecystectomy with SA were asked about their opinion regarding this issue in a study and all surgeons agreed that this anesthetic technique was satisfactory, abdominal relaxation was adequate for surgery, and they had no problems related to the anesthetic technique^(7)^. However, right shoulder pain was also reported as a disadvantage of SA, which resulted in increased intraabdominal pressure limiting laparoscopic exploration^(7)^. Nevertheless, laparoscopic surgery was performed uneventfully after intraoperative relief of the shoulder pain. We observed similar responses according to the answers of surgeons’ questionnaire in our study. Abdominal relaxation was expressed as “good”, there were generally no technical problems arising from SA, and there was no surgical difference between patients under SA and GA.

Reactive oxygen species (ROS) are produced in metabolic and physiologic pathways. Harmful oxidative reactions may occur in organisms, which cannot extinguish ROS via enzymatic and non-enzymatic mechanisms. In specific conditions, an increase in oxidants and decrease in antioxidants cannot be prevented and the balance between oxidative and antioxidative equilibrium changes in favor of an oxidative state^(8)^. Trauma, sepsis, and surgical injury (especially ischemia-reperfusion injury) are related to increased ROS production^(4,27)^. Many oxidant molecules exist in blood, which prevent and/or inhibit the harmful effects of ROS. The effects of antioxidant effects of plasma are additive and the measurement of total antioxidant status specifies the antioxidative status of plasma. Cooperation of various antioxidants in human plasma ensures protection against oxidative stress.

On the contrary, the increase of intraabdominal pressure, which depends on pneumoperitoneum, may cause splanchnic ischemia^(28,29)^. After deflation of the abdomen, intraabdominal pressure and splanchnic blood pressure normalization and reperfusion occur. Less surgical trauma and minimal tissue injury associated with laparoscopic procedures may be suggested to cause less oxidative stress. However, clinical outcomes of oxidative stress due to ischemia-reperfusion during laparoscopic procedures are still unclear^(27)^.

Intraabdominal pressure increases to 10-15 mm Hg during the induction of pneumoperitoneum, and this pressure level is significantly higher than in the portal system (7-10 mm Hg). Previous human studies showed that pneumoperitoneum created a prominent decrease in gut perfusion and hepatic microcirculation^(30)^.

Deflation of pneumoperitoneum results with a decrease in intraabdominal pressure and an increase splanchnic perfusion. Thus, laparoscopic surgery may present an ischemia-reperfusion model^(31)^. The term ‘ischemia-reperfusion’ includes the consumption of energy sources of cells and the accumulation of free radicals in the circulation due to high levels of O_2_ following reperfusion. Oxygen-originated cytotoxic products may cause the current circumstance and free oxygen radicals play a critical role in injury brought about by ischemia-reperfusion. The main cause of toxicity in different tissues is this production of ROS, which lead to an inflammatory response and tissue injury by activating various mediators^(4)^. High levels of hydrogen peroxide and other peroxides diffuse to plasma in physiologic or pathologic conditions^(9)^. The ratio of total peroxide to total antioxidant potential is called OSI, which is an indicator of the level of oxidative stress^(10)^. In our study, OSI was higher in the GA group than in the SA group, even though sevoflurane was used instead of halothane, which disrupts lipid peroxidation and antioxidant defense. However, this difference was not statistically significant. Free radicals are probably produced in laparoscopic procedures and they attack lipid molecules, and they interact with low-molecular-weight antioxidants in plasma. However, the results of our study reveal that similar TAC levels are reached in gynecologic laparoscopic surgery under SA and GA.

Our study is, as far as we know, the first to evaluate the effects of SA and GA on oxidative-antioxidative status in gynecologic laparoscopic surgery. Monitoring of TAC, TOL, and OSI may become important biochemical indicators in future clinical settings in terms of evaluating and preventing oxidative cell and tissue injury during laparoscopic surgery. In light of our findings, we can speculate that SA causes no more oxidative stress in gynecologic laparoscopy cases than GA. Evaluation of TAC, TOL and OSI with other clinical parameters may ensure better management of surgical treatment, especially in patients with unexplained infertility.

### Study Limitations

Among the limitations of our study are the relatively small sample size (although this is a pilot study) and subjective nature of the questionnaire.

## Conclusion

In the era of minimally invasive surgical approaches, anesthetic techniques should decrease the impact of surgical stress to organisms and the postoperative complications arising from anesthesia and surgery, and help patients and surgeons feel more comfortable. Also, one can comment on the protective benefits of SA compared with GA for all operating room staff including surgeons, anesthetists, and others in the “new-normal era” after the coronavirus 2019 outbreak.

SA may become a reliable and less invasive alternative to GA in gynecologic laparoscopy when equivalent and even advantageous features in terms of hemodynamic and respiratory parameters, patient and surgeon satisfaction, as well as total oxidant, antioxidant levels, and OSIs are considered. The need for further large-scale randomized prospective studies is evident to provide convincing evidence for the routine use of this safe and patient-friendly technique.

## Figures and Tables

**Table 1:**
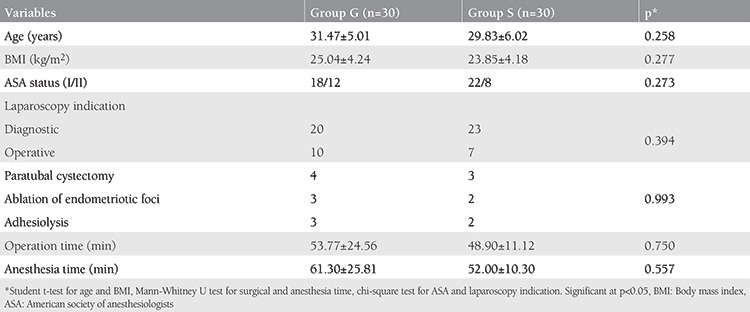
Demographic and operation characteristics of the patients

**Table 2:**
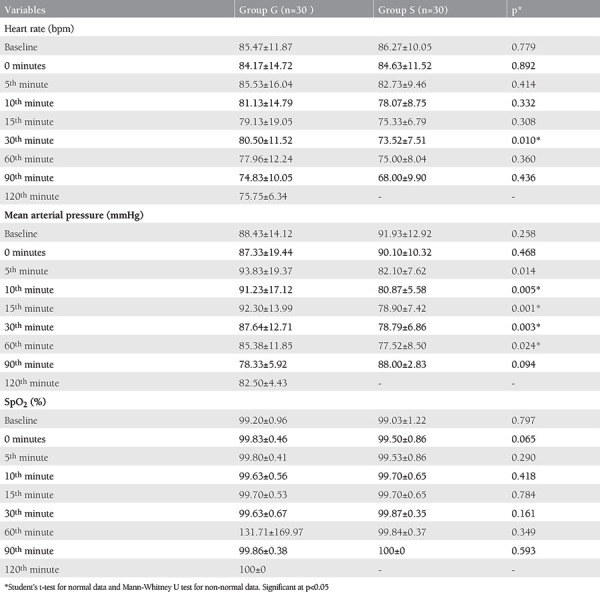
Hemodynamic variables of the patients

**Table 3:**
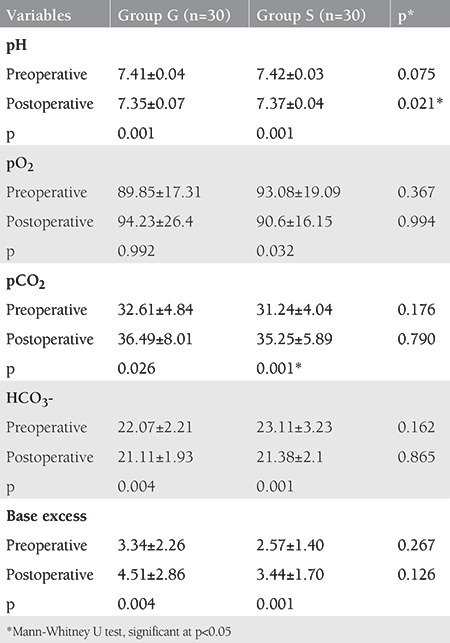
Arterial blood gas parameters of the patients

**Table 4:**
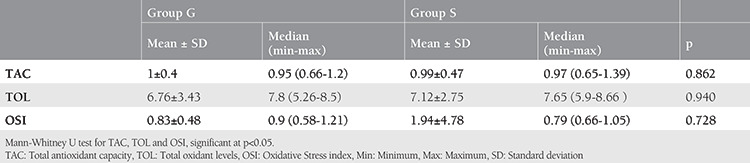
Total antioxidant capacity, total oxidant levels and oxidative stress index of the patients
